# Surgical management and outcomes following atypical subtrochanteric femoral fractures − results from a matched-pair analysis of the registry for geriatric trauma of the German Trauma Society

**DOI:** 10.1007/s00402-024-05297-3

**Published:** 2024-04-20

**Authors:** Christopher Bliemel, Robert Birkelbach, Tom Knauf, Bastian Pass, Benjamin Craiovan, Carsten Schoeneberg, Steffen Ruchholtz, Martin Bäumlein, on behalf of the AltersTraumaRegister DGU

**Affiliations:** 1 Center for Orthopaedics and Trauma Surgery, University Hospital Marburg, 35043 Baldingerstrasse, Marburg, Germany; 2 Academy for Trauma Surgery, AUC, Munich, Germany; 3 Department of Orthopedic and Emergency Surgery, Alfried Krupp Hospital, Essen, Germany; 4 Working Commitee on Geriatric Trauma Registry (AK ATR) of the German Trauma Society (DGU), Berlin, Germany; 5 Philipps University of Marburg, Marburg, Germany

**Keywords:** Atypical subtrochanteric femoral fracture, Hip fracture, Outcome, Mortality, Mobility, AltersTraumaRegister DGU®

## Abstract

**Background and objectives:**

The outcomes of patients with atypical subtrochanteric fractures (ASFs) remain unclear. Data from a large international geriatric trauma registry were analysed to examine the outcome of patients with ASFs compared to patients with typical osteoporotic subtrochanteric fractures (TSFs).

**Materials and methods:**

Data from the Registry for Geriatric Trauma of the German Trauma Society (Deutsche Gesellschaft für Unfallchirurgie [DGU]) (ATR-DGU) were analysed. All patients treated surgically for ASFs or TSFs were included in this analysis. Across both fracture types, a paired matching approach was conducted, where statistical twins were formed based on background characteristics sex, age, American Society of Anesthesiologists (ASA) score and walking ability. In-house mortality and mortality rates at the 120-day follow-up, as well as mobility at 7 and 120 days, the reoperation rate, hospital discharge management, the hospital readmission rate at the 120-day follow-up, health-related quality of life, type of surgical treatment and anti-osteoporotic therapy at 7 and 120 days, were assessed as outcome measures using a multivariate logistic regression analysis.

**Results:**

Amongst the 1,800 included patients, 1,781 had TSFs and 19 had ASFs. Logistic regression analysis revealed that patients with ASFs were more often treated with closed intramedullary nailing (RR = 3.59, *p* < 0.001) and had a higher probability of vitamin D supplementation as osteoporosis therapy at 120 days (RR = 0.88, *p* < 0.002). Patients with ASFs were also more likely to live at home after surgery (RR = 1.43, *p* < 0.001), and they also tended to continue living at home more often than patients with TSFs (RR = 1.33, *p* < 0.001). Accordingly, patients with TSFs had a higher relative risk of losing their self-sufficient living status, as indicated by increased rates of patients living at home preoperatively and being discharged to nursing homes (RR = 0.19, *p* < 0.001) or other hospitals (RR = 0.00, *p* < 0.001) postoperatively.

**Conclusions:**

Surgical treatment of ASFs was marked by more frequent use of closed intramedullary fracture reduction. Furthermore, patients with ASFs were more likely to be discharged home and died significantly less often in the given timeframe. The rate of perioperative complications, as indicated by nonsignificant reoperation rates, as well as patient walking abilities during the follow-up period, remained unaffected.

**Supplementary Information:**

The online version contains supplementary material available at 10.1007/s00402-024-05297-3.

## Introduction

The World Health Organization (WHO) ranks osteoporosis among the ten most common and important diseases worldwide, as it contributes to a tremendous number of hip fractures, amongst other fractures [1]. In affected patients, such fractures are associated with an extremely high mortality rate and a devastating loss of function [2–9].

**Fig. 1:**
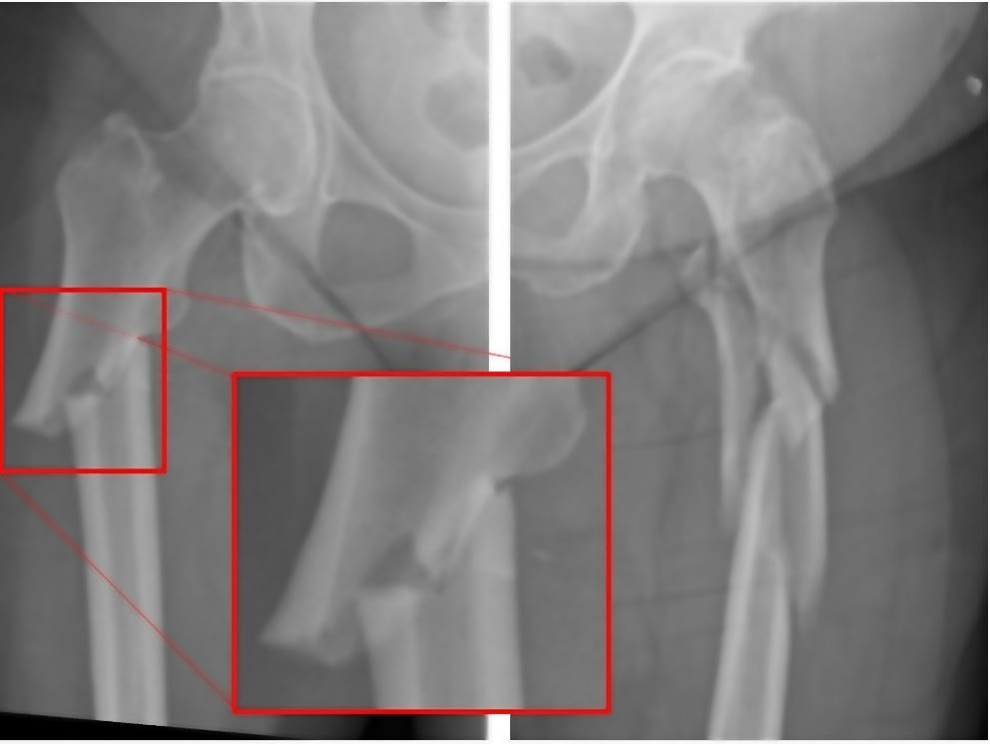
Illustration of the two different types of subtrochanteric femoral fractures. At the right femur, an atypical fracture pattern is demonstrated (ASF). The atypical fracture pattern is characterized by a simple transverse or short oblique fracture. In the red rectangle, the fracture region is enlarged. The enlarged view shows the typical thickening of the cortex.The left femur shows a subtrochanteric fracture with a typical osteoporotic fracture pattern (TSF)

Approximately eight million people are estimated to be affected in Germany alone [10]. In this context, bisphosphonates are currently the major class of drugs used for osteoporosis therapy both in Germany and worldwide [11, 12]. While aiming to reduce the risk of osteoporotic fractures, bisphosphonates are also associated with atypical fractures themselves. Such atypical femoral fractures commonly occur in the subtrochanteric and diaphyseal region of the femur and are frequently caused by bisphosphonates, as these substances suppress bone turnover and inhibit the targeted remodelling of osseous microdamage [13]. But also, other medical causes like a prolonged intake of e.g. monoclonal antibodies are associated with atypical femoral fractures. Studies have shown that denosumab, similar to oral alendronate, may confer a risk of ASF through its effect on targeted bone remodeling [14]. In this context, long-term use of denosumab, especially for more than 3.5 years has been identified as risk factor for the development of atypical subtrochanteric fractures (ASFs) in cancer patients [15]. Figure 1 illustrates such an ASF, which is characterized by a fracture originating in the lateral cortex with a simple transverse or a short oblique fracture line in areas of a thickened corticalis in contrast to an osteoporosis-related typical subtrochanteric fracture (TSF) [16]. According to the American Society for Bone and Mineral Research (ASBMR) task force, apart from the morphological aspects of these fractures, atypical femoral fractures are characterized by a minimal or even no history of trauma, no or only a minimal debris zone and a localized periosteal or thickening of the lateral cortex [17]. The absolute risk for the occurrence of such atypical fractures is low, with an incidence of 1.8 per 100,000 patient-years after 2 years of therapy. Nevertheless, the probability for atypical fractures of the femur increases with an increase in the length of the treatment, leading up to 113 fractures per 100,000 patient-years at a bisphosphonate exposure period of 8–10 years [13, 17–19].

Despite this risk for atypical fractures, the benefits of antiresorptive therapy with bisphosphonates by far outweigh these side effects. In this regard, a study on 1.8 million patients demonstrated that over a 5-year period, 162 fragility fractures of the spine, hip and forearm could be prevented for each atypical femoral fracture [19].

Against the background of the abovementioned disturbance in bone metabolism, along with a decreased heterogeneity of minerals, lowered toughness in bone and other effects [17], it can be assumed that bisphosphonates and also monoclonal antibodies might also have a negative effect on fracture healing and therefore on the outcomes of patients affected by atypical femoral fractures.

Currently, the literature on this topic remains limited and contradictory. Some studies report similarities between the two groups of patients, especially in terms of the 30-day mortality rate [20], the rate of reoperations [21] and the existence of certain comorbidities, such as chronic liver and pulmonary diseases [11].

On the other hand, discordant findings are found in other studies, including the age of the patients affected [22], the previous occurrence of a hip fracture, the history of systemic glucocorticoid intake and the existence of certain comorbidities such as depression [23] and diabetes mellitus [21].

To provide more clarity on this controversial topic, the data of the Registry for Geriatric Trauma (AltersTraumaRegister DGU® [ATR-DGU]) of the German Trauma Society (Deutsche Gesellschaft für Unfallchirurgie [DGU]) were analysed as part of the present study.

It was hypothesized that, compared to TSFs, atypical fracture patterns associated with bisphosphonate or monoclonal antibody intake would lead to increased rates of perioperative complications and prolonged reconvalescence among patients with ASFs.

## Materials and methods

### Data and methods

This study is based on the ATR-DGU (https://www.alterstraumaregister-dgu.de, data version year 2023). The ATR-DGU is a register where the data are collected in a prospective, standardized, pseudonymized manner and come from multiple geriatric trauma centres. The focus of the ATR-DGU is on geriatric trauma patients who suffer from hip fractures as well as periprosthetic and peri-implant femoral fractures [24, 25]. Patients included in the ATR-DGU must be at least 70 years of age.

The Academy for Trauma Surgery (AUC - Akademie der Unfallchirurgie GmbH) provides the infrastructure for documentation, data management, and analysis. The scientific management is conducted by the Working Committee on Geriatric Trauma Registry (AK ATR) of the German Trauma Society (DGU). All certified Centers for Geriatric Trauma (AltersTraumaZentrum DGU^®^) are required to participate in the ATR-DGU. These centres submit pseudonymized patient data through a web-based application into a central database. The standard documentation sheet consists of approximately 160 data fields for each patient.

Currently, the ATR-DGU receives contributions from hospitals in three countries: Germany, Switzerland and Austria. The total dataset includes more than 62,000 cases from nearly 160 hospitals thus far. The data used in this study were collected from preoperative records from 2016 to 2022 and included data collected at the time of hospital admission, during surgery, one week after surgery and optionally at 120 days postoperatively [26].

The ATR-DGU data contain detailed information on demographics, preoperative residential and health status, comorbidities, fracture pattern, time course, relevant medication history and outcomes for each individual patient. Approval for scientific data analysis is obtained through a peer-review process in accordance with the publication guidelines specified by the AK ATR. This study adhered to the publication guidelines of the ATR-DGU and is registered as ATR-DGU project ID ATR-2021-006.

### Aim of the study and outcome variables

The aim of the study was to gain a better understanding of ASFs, which are very rare and can occur as a side effect of bisphosphonate and also monoclonal antibody osteoporosis therapy. Patients were classified as having an ASF if their fracture met the ASBMR task force criteria [27]. To be considered atypical, the location of the fracture had to be below the lesser trochanter, and four out of five major features had to be present: (a) the fracture was associated with minimal or no trauma (fall from standing height or less); (b) the fracture line originated at the lateral cortex and could be transverse in orientation or oblique as it progressed medially; (c) complete fractures extending to both cortices; (d) the fracture was noncomminuted or minimally comminuted; and (e) the fracture showed localized periosteal or endosteal thickening of the lateral cortex [27].

The outcome variables determined for this analysis were mortality during the acute hospital stay and until the 120-day follow-up as well as mobility and the reoperation rate after 7 and 120 days, hospital discharge management, the hospital readmission rate until the 120-day follow-up and patient quality of life according to the EQ-5D score at 7 and 120 days after the operation. The variables walking ability and residential status were recoded to be binary, as shown in Tables 1 and 2.

**Table 1:** Univariable analysis of unmatched, acute care data on geriatric trauma patients with typical and atypical subtrochanteric hip fractures

Parameter		Typical subtrochanteric fracture	Atypical subtrochanteric fracture	*p*-value
Number of patients		1781	19	
Gender	Male Female	443 (25.7%) 1278 (74.3%)	4 (21.1%) 15 (78.9%)	0.840
Patient age (year) Mean (sd)		84 (78; 90)	81 (75; 87)	< 0.001
ASA score	1 2 3 4 5	18 (1.0%) 373 (21.7%) 1213 (70.5%) 113 (6.6%) 4 (0.2%)	0 (0.0%) 7 (36.8%) 12 (63.2%) 0 (0.0%) 0 (0.0%)	0.460
Prefracture walking ability	Independent without walking aids Ability to walk outside with a walking stick or crutch Ability to walk outside with two crutches or a walker Certain walking ability in the apartment, but outside only with an assistant No functional walking ability	636 (37.0%) 210 (12.2%) 560 (32.5%) 255 (14.8%) 60 (3.5%)	7 (36.8%) 0 (0.0%) 6 (31.6%) 6 (31.6%) 0 (0.0%)	0.160
Specific drug therapy for osteoporosis at the 7th postoperative day	Yes No	240 (57.0%) 181 (43.0%)	13 (81.2%) 3 (18.8%)	0.360
Vitamin D therapy for osteoporosis at the 7th postoperative day	Started Continued Recommended	1090 (69.3%) 459 (29.2%) 24 (1.5%)	14 (77.8%) 4 (22.2%) 0 (0.0%)	0.685
Prefracture residential status	At home \ Assisted living facility Nursing home Hospital \ Inpatient facility	1388 (82.0%) 288 (17.0%) 16 (1.0%)	18 (94.7%) 1 (5.3%) 0 (0.0%)	0.702
Surgical treatment with intramedullary nail	Yes No	1640 (99.8%) 3 (0.2%)	13 (100.0%) 0 (0.0%)	1.000
Intramedullary nail – type of reduction	Not specified Closed reduction Open reduction without cerclage Open reduction with cerclage	421 (27.7%) 247 (16.3%) 698 (45.9%) 155 (10.2%)	2 (16.7%) 7 (58.3%) 3 (25.0%) 0 (0.0%)	0.001
EQ-5D index at the 7th postoperative day		Mean = 0.44, SD = 0.30 *n* = 1290	Mean = 0.55, SD = 0.33 *n* = 13	< 0.001
Reoperation within acute hospital stay	Yes No	54 (3.8%) 1374 (96.2%)	2 (12.5%) 14 (87.5%)	0.252
Discharge from hospital	At home Nursing home Inpatient stay	332 (22.0%) 434 (28.8%) 741 (49.2%)	5 (27.8%) 6 (33.3%) 7 (38.9%)	0.805
7-day mortality rate	Alive Dead	1683 (97.8%) 38 (2.2%)	19 (100.0%) 0 (0.0%)	1.000
7 day walking ability	Independent without walking aids Ability to walk outside with a walking stick or crutch Ability to walk outside with two crutches or a walker Certain walking ability in the apartment, but outside only with an assistant No functional walking ability	5 (0.3%) 113 (6.8%) 342 (20.6%) 719 (43.4%) 478 (28.8%)	0 (0.0%) 2 (11.1%) 3 (16.7%) 8 (44.5%) 5 (27.8%)	0.759

**Table 2:** Univariable analysis of unmatched, 120-day follow-up data on geriatric trauma patients with typical and atypical subtrochanteric hip fractures

Parameter		Typical subtrochanteric fracture	Atypical subtrochanteric fracture	*p*-value
Number of patients		1516	18	
Ability to walk	Without aid With walking stick or crutch With two crutches or a rollator Certain ability to walk indoor Not possible	42 (7.7%) 74 (13.5%) 239 (43.6%) 112 (20.4%) 81 (14.8%)	2 (28.6%) 1 (14.3%) 3 (42.9%) 1 (14.3%) 0 (0.0%)	0.290
Residential status	At home \ Assisted living facility Nursing home Hospital \ Inpatient facility	371 (72.0%) 130 (25.2%) 14 (2.7%)	6 (85.7%) 1 (14.3%) 0 (0.0%)	0.669
120-day mortality	Alive Dead	1376 (91.1%) 154 (8.9%)	18 (100%) 0 (0%)	0.347
Changes in living situation	Prefracture living at home and still living at home Prefracture living at home has changed to nursing home Prefracture living at home has changed to other inpatient facility	357 (83.8%) 58 (13.6%) 11 (2.6%)	6 (85.7%) 1 (14.3%) 0 (0.0%)	0.911
Specific drug therapy for osteoporosis	Yes No	286 (85.4%) 49 (14.6%)	3 (60%) 2 (40%)	0.344
Vitamin D therapy for osteoporosis	Yes No	261 (55.2%) 212 (44.8%)	3 (60%) 2 (40%)	1.000
Readmission to hospital during follow-up	Yes No	35 (5.2%) 632 (94.8%)	1 (12.5%) 7 (87.5%)	0.908
Reoperation during follow-up	Yes No	28 (4.9%) 542 (95.1%)	1 (16.7%) 5 (83.3%)	0.710
EQ-5D index		Mean = 0.66, SD = 0.28, *n* = 424	Mean = 0.66, SD = 0.22, *n* = 5	0.990

### Subsample selection

The population eligible for inclusion in the ATR-DGU are patients who are at least 70 years old and at most 110 years old and either undergo hip fracture surgery or have peri-implant or periprosthetic fractures of the femur. Out of this sample, the only relevant cases for this study were patients who had either nonpathological TSFs (*n* = 1781) or ASFs (*n* = 19). Therefore, a total of 1,800 patients were eligible for this study. As multiple imputation of the missing data was unsuitable due to the small treatment group size, we used listwise deletion. The sample sizes of each model can be inferred from the descriptive tables.

### Analysis approach

In the descriptive analyses, categorical data are presented as counts and percentages, and continuous variables are shown as the mean and standard deviation.

To aid in drawing causal conclusions, this study employed a paired matching approach, where statistical twins were formed based on background characteristics, sex, age, American Society of Anesthesiologists (ASA) score and walking ability at the time of admission.

Given the extreme rarity of ASFs, we choose a full optimal matching approach. This approach provided the best balance of all approaches we tried while ensuring that no data were discarded in the matching process, in addition to missing values. Full optimal matching finds at least one match for each control and at least one match for each member of the treatment group. This approach minimizes the distances between the members of the treatment and control groups of each subclass.

After matching was performed, we employed linear regression models for continuous outcome variables, general linear models with a quasibinomial link function for binary outcome data and multinomial logistic regression for categorical outcome variables with more than 2 categories. All the models were weighted with the weights we obtained from the matching procedure. A quasibinomial distribution is similar to a binomial distribution but has an extra parameter to model variation that cannot be explained through the binomial distribution alone. This was necessary due to the small group size of patients with ASFs. All models include interaction effects of the fracture variable and the matching variables so that we arrived at the pure main effect.

Then, we performed g-computation to estimate the average treatment effect. This was done by producing predicted values for each patient where the independent variable was set to 0 or 1, respectively, which were the potential patient outcomes. Afterwards, the mean of both groups was calculated over the entire sample, and the contrast was calculated. The effect size, *p* value and 95% confidence intervals are reported. Given the small treatment group size, we did not put too much emphasis on inferential statistics.

The model “discharge from hospital” used a newly coded variable, which showed a difference in the patient’s prefracture and postrelease living situation. The average treatment effect on the categorical data models is expressed in terms of average relative risks for TSF vs. ASF.

## Results

**Fig. 2:**
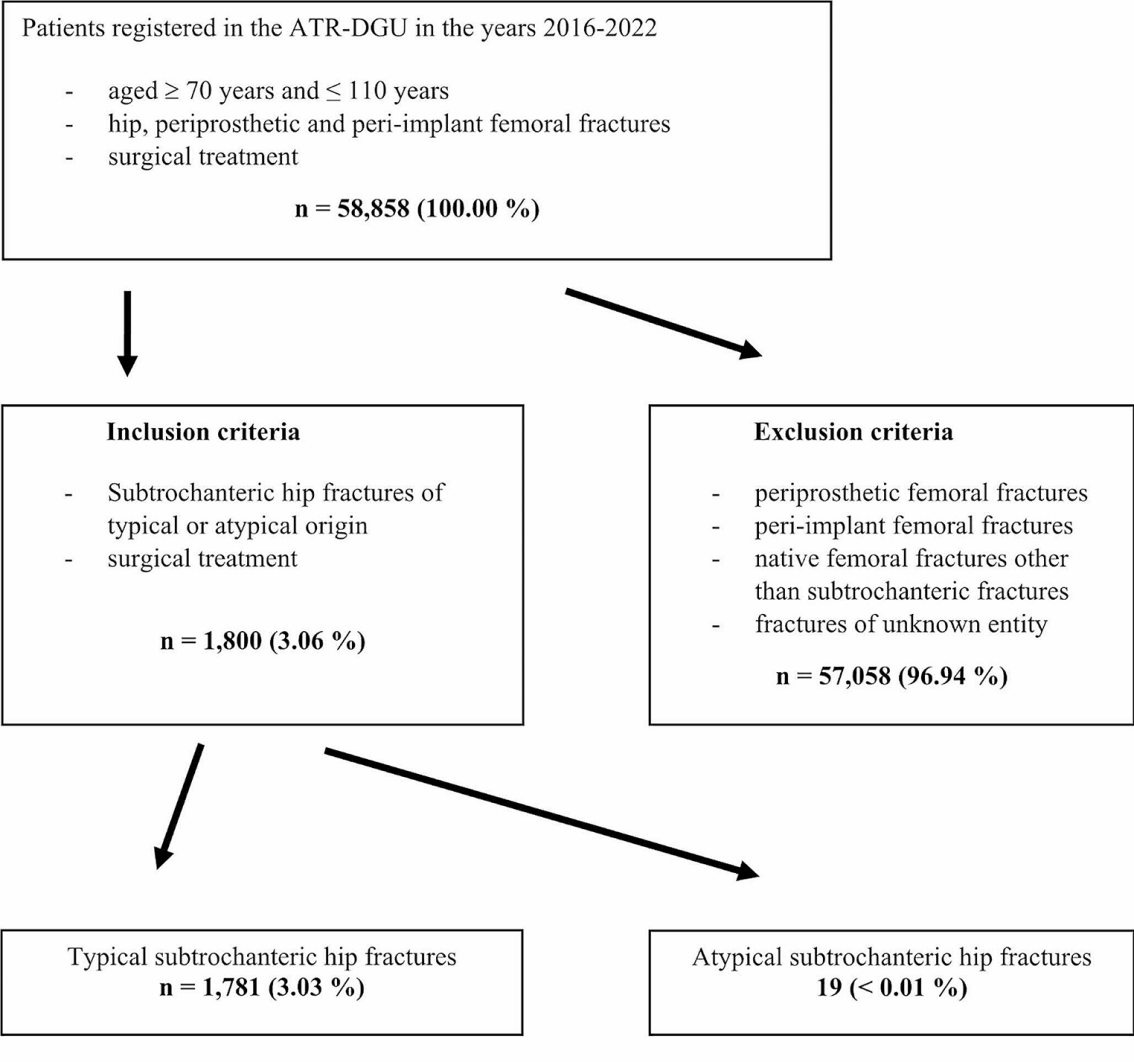
Flow sheet of the included population

A total of 1,800 subtrochanteric femoral fractures in geriatric trauma patients were included in this study. Of these fractures, 1,781 were TSFs, and 19 were ASFs (Fig. 2).

Descriptive baseline data analysis in terms of the fracture origin, whether typical or atypical, is demonstrated in Table 1. Analysis of patient age revealed that patients in both groups were predominantly in their late 70s and 80s, with values for the 25th and 75th quartiles ranging from 78 years to 90 years for patients suffering from TSFs and from 75 years to 87 years for patients suffering from ASFs, respectively. Through this, patients presenting with ASFs were not only significantly younger (*p* < 0.001) in age but also had a significantly increased quality of life according to the EQ-5D index at the 7th postoperative day (*p* < 0.001). No differences were seen in terms of sex distribution, with female patients being predominantly represented in both groups (74.5% vs. 78.9%; *p* = 0.840) in terms of the estimated perioperative risk as indicated by the ASA score (*p* = 0.460), prefracture walking ability (*p* = 0.160), anti-osteoporotic drug therapy (*p* = 0.360), vitamin D supplementation (*p* = 0.685), prefracture residential status (*p* = 0.702), rate for reoperation during the acute hospital stay (*p* = 0.252), discharge from the hospital (*p* = 0.805), 7-day mortality rate (*p* = 1.000) and walking ability (*p* = 0.759). Concerning surgical treatment, intramedullary nailing was predominant in both groups (*p* = 1.000). Nevertheless, concerning the type of reduction, ASFs were treated with closed reduction significantly more often, whereas TSFs were treated more often with open reduction, with or without the application of a wire cerclage (*p* = 0.001).

The univariable analysis 120-day follow-up data are shown in Table 2. None of the parameters assessed showed statistical significance.

To further evaluate the data of TSF vs. ASF regression analyses (linear, quasibinomial and multinomial) were performed, adjusted for sex, patient age, ASA score, fracture type and prefracture walking ability and weighted by the weights derived from the matching procedure (Table 3). Patients with ASFs were more often treated with closed intramedullary nailing (RR = 3.59, *p* < 0.001) and had a higher probability of receiving specific anti-osteoporotic drug therapy after 7 (RR = 1.25, *p* < 0.001) but not at 120 days (RR = 1.59, p 0.140) than patients with TSFs. The probability of starting vitamin D intake as osteoporosis therapy was also significantly increased for patients with ASFs at 7 days (RR = 1.43, *p* < 0.001) and 120 days (*p* = 0.002).

**Table 3:** Regression analysis of TSF vs. ASF. Analysis is adjusted for sex, patient age, ASA score, fracture type and prefracture walking ability. The model “discharge from hospital” is adjusted to the prefracture living situation. At estimates greater than 1, the probability of the particular item is more likely to be associated with ASF. Estimates close to 0 indicate association with TSF

Influence of the fracture entity on…		*N*	estimate	95% CI	*p*-value
**Acute phase**					
Walking ability after 7 days *		1675	1.12	[0.82; 1.56]	0.660
Death during stay in the acute hospital 7 days *		1740	0.00	[0.00; 0.00]	< 0.001
Equation 5D index 7 days ~		1303	0.06	[-0.01; 0.14]	0.090
Intramedullary nail ^+^	Closed reduction	1533	3.59	[2.93; 4.39]	< 0.001
Intramedullary nail ^+^	Open reduction with cerclage	1533	0.00	[2.85e-187; 8.19e + 176]	0.960
Intramedullary nail ^+^	Open reduction without cerclage	1533	0.59	[0.36; 0.97]	0.040
Specific anti-osteoporotic therapy at 7 days *		1440	1.25	[1.09; 1.43]	< 0.001
Specific anti-osteoporotic therapy at 7 days *	Begun	39	0.01	[5.49e-02; 3.87e + 05]	0.216
Specific anti-osteoporotic therapy at 7 days *	Recommended	39	8.95e-24	[1.41e-36; 5.68e-11]	< 0.001
Specific anti-osteoporotic therapy at 7 days *	Changed	39	1.95e-03	[0.00e + 00; Inf]	0.00
Specific anti-osteoporotic therapy at 7 days *	Continued	39	2.63e-49	[7.22e-61; 9.57e-38]	< 0.001
Vitamin D intake for osteoporosis therapy at 7 days *	Begun	81	1.43	[1.18; 1.72]	< 0.001
Vitamin D intake for osteoporosis therapy at 7 days *	Recommended	81	0.02	[0.01; 0.04]	< 0.001
Vitamin D intake for osteoporosis therapy at 7 days *	Continued	81	0.00	[9.82e-87; 2.46e-84]	< 0.001
Domicile ^+^	At home \ Assisted living facility	529	1.43	[1.35; 1.51]	< 0.001
Domicile ^+^	Nursing home	529	0.17	[0.15; 0.19]	< 0.001
Domicile ^+^	Hospital \ Inpatient Facility	529	0.00	[0.00; 0.00]	< 0.001
Discharge from hospital ^+^	Prefracture living at home and still living at home	433	1.33	[1.25; 1.41]	< 0.001
Discharge from hospital ^+^	Prefracture living at home has changed to nursing home	433	0.19	[0.16; 0.22]	< 0.001
Discharge from hospital ^+^	Prefracture living at home has changed to other inpatient facility	433	0.00	[0.00; 0.00]	< 0.001
**120 days follow-up**					
Walking ability after 120 days *		555	1.17	[0.57; 2.42]	0.660
Death during follow up *		1740	0.00	[0.00; 0.00]	< 0.001
Equation 5D index 120 days ~		429	0.02	[-0.05; 0.10]	0.530
Reoperation during follow-up *		576	2.91	[0.36; 23.57]	0.320
Specific anti-osteoporotic therapy at 120 days *		340	1.59	[0.86; 2.91]	0.140
Vitamin D intake for osteoporosis therapy at 120 days *		478	0.88	[0.81; 0.95]	0.002

*logistic regression; ~ linear regression; ^+^ multinomial regression

Patients with ASFs had a higher chance of living at home after surgery (RR = 1.43, *p* < 0.001), and they tended to continue living at home more often than patients with TSFs (RR = 1.33, *p* < 0.001). On the other hand, preoperatively, patients with TSFs more often lived in nursing homes (RR = 0.17, *p* < 0.001) or inpatient facilities (RR = 0.00, *p* < 0.001). Additionally, postoperatively, patients with TSFs had a higher relative risk of losing their self-sufficient living status, as indicated by increased rates of patients living at home preoperatively and being discharged to nursing homes (RR = 0.19, *p* < 0.001) or other hospitals (RR = 0.00, *p* < 0.001) postoperatively.

Health-related quality of life, as indicated by the EQ-5D index, remained nonsignificant between the two fracture types after both 7 and 120 days. Additionally, patients’ walking ability after 7 and 120 days as well as the rate of reoperation, dependent on both fracture entities, remained unaffected. None of the patients with ASFs died in this study; hence, the relative mortality risk was 0 at the 7th postoperative day and the 120-day follow-up.

## Discussion

This study aimed to analyse the impact of ASFs compared to TSFs. An ASF pattern did not lead to increased rates of perioperative complications or prolonged reconvalescence, as indicated by insignificant rates in reoperation and walking abilities during the 120-day follow-up period (*p* = 0.320; *p* = 0.660). Rather, it was shown that patients with ASFs were more likely to be discharged home (*p* < 0.001), died significantly less often (*p* < 0.001) and were more often treated with closed intramedullary reduction (*p* < 0.001) than patients with TSFs.

Through a retrospective comparative observational study of patients aged ≥ 55 years, *Kharazmi et al.* showed that, in contrast to ordinary subtrochanteric and femoral shaft fractures, atypical femoral fractures were not associated with excess mortality [28]. Additionally, in a single centre study on 462 subtrochanteric femoral fractures, *Gani et al.* reported no evidence for increased mortality in patients with ASFs compared to those with TSFs [21]. Therefore, the results of the present study also confirm these previous results in a more geriatric patient cohort aged 70 years and older. Our results are in line with those of *Gani et al.*, not only in terms of mortality but also in terms of comparable rates of reoperation between patients suffering from TSFs and ASFs [21]. Additionally, comparable results have been published by *Khow et al.* [29]. By conducting a retrospective matched cohort study on 710 hip fractures in Australia, they recognized equal outcomes in terms of mortality, mobility and the reoperation rate in atypical femoral fracture patients when compared to typical femoral fracture patients. This contradicts some previous studies that have indicated generally poorer outcomes for patients with atypical femoral fractures [30, 31]. Nevertheless, our study provides further evidence that patients with ASFs do not neccesarily have worse outcomes, as indicated by insignificant rates of repeat surgical interventions compared to patients suffering from TSFs.

*Spanyer et al.* investigated health-related quality of life outcomes in patients after surgical treatment for atypical femur fractures using a multicentre retrospective cohort study model [32]. By applying the Short Form 36, version 2 (SF-36 v.2) health survey, they found mid-term patient-reported quality of life outcomes to be similar among women who sustained an atypical femoral fracture compared to a cohort of patients with typical femoral diaphyseal fractures. Additionally, in our more elderly patient cohort, the EQ-5D index at 7 and 120 days after surgery remained nonsignificant between both groups of patients. Furthermore, our study results could show that health-related quality of life outcomes are also unaffected in a mixed-sex patient collective.

In terms of surgical fracture treatment, our study results demonstrate that patients with ASFs were significantly more often treated with closed reduction, whereas patients with TSFs were significantly more often treated with open reduction without cerclage. Older publications have mainly reported on plate osteosynthesis for sole fixation of pathological femur fractures. However, these publications also noted increased rates of osteosynthesis failure [29, 30, 33]. Therefore, there has been a change in the paradigm in which intramedullary nails are now the standard in the treatment of ASFs, not only in terms of more favourable biomechanical loading properties with on-axis fixation and collinear strain [20, 34–36] but also in terms of fracture healing [37, 38]. For this reason, the results of our study confirm the results from the previous literature.

Regarding medical therapy for ASFs, the results of the present register analysis revealed that vitamin D intake for osteoporosis therapy was significantly more often begun at 7 days after surgery in ASF patients, whereas it was significantly more often recommended or continued in TSF patients. This recommendation of the start or continuation of vitamin D supplementation apparently also led to TSF patients taking vitamin D more frequently even at 120 days after surgery. Seven days after surgery, specific anti-osteoporotic therapy was recommended significantly more frequently for TSF patients. The fact that vitamin D intake was started in ASF patients earlier compared to TSF patients is somehow surprising. Thus, the cause of an ASF is due to an existing osteoporosis therapy, which generally consists of a basis therapy (supplementation of vitamin D) and an additional specific medication (e.g. bisphosphonates). Therefore, the authors believe that the observed intake of vitamin D in ASF patients is due to an already existing osteoporosis therapy. In how far a specific treatment with antiresorptive drugs was stopped or if a switch towards an osteanabolic drug was initiated - such as e.g. teriparatide - remains unclear due to limitations in the study design and the documentation sheet respectively.

According to the ASBMR report, antiresorptive treatment should be discontinued if a fracture occurs. Four to six weeks after surgical therapy, treatment with teriparatide can be started, taking into account contraindications [27]. *Dell et al.* observed that the risk of suffering a contralateral fracture after the diagnosis of an atypical femoral fracture could be reduced by 53% when bisphosphonate intake was stopped [19]. Additionally, *Schilcher et al.* demonstrated a reduction in the relative risk of developing an ASF on the opposite side after discontinuation of bisphosphonate therapy [23]. However, it must be considered whether discontinuing therapy in these patients could lead to rebound fractures due to underlying osteoporosis.

Also denosumab, an antibody that blocks the formation of osteoclasts, has been associated with the potential to cause ASFs, similar to bisphosphonates [14]. The risk of atypical fractures may be influenced by denosumab’s mechanism of action, which disrupts targeted bone remodeling by blocking RANKL, a molecule crucial for osteoclast function [14]. Studies have reported cases of atypical femoral fractures in patients under denosumab treatment, especially after long-term use, with some patients developing recurrent fractures despite therapy changes [15, 39]. The incidence of atypical femoral fractures is a concern in patients receiving denosumab for various conditions, including osteoporosis and bone metastasis [15, 40, 41].

Preoperatively, patients with TSFs significantly more often lived in nursing homes or inpatient facilities, whereas ASF patients more often lived independently before their fracture. Additionally, our discharge analysis from the hospital revealed that ASF patients were more often discharged home, whereas TSF patients lost their self-independence significantly more often. This is indicated by increased rates of discharge to nursing homes and other inpatient facilities, particularly among patients who lived at home before suffering their hip fracture. Therefore, the results of the present study contradict some previous research from *Subramanian and Parker* [42], *Davenport et al.* [20] and *Khow et al.* [29], who reported comparable discharge locations for patients with typical and atypical femoral fractures. A possible explanation for this circumstance might be the fact that in these previous publications, a multivariate data analysis was not performed. While in the univariate analysis of our data, there were also only insignificant values between both fracture types with regard to discharge management (Table 1), a significant difference could only be demonstrated after adjustment for certain confounding parameters.

## Limitations

Since the present analysis is based on registry data, some limitations must be taken into account. First, it must be mentioned that only well-designed randomized trials can demonstrate causality, whereas registry analyses, such as the present study, can only describe relationships. Therefore, our findings must be interpreted with some caution.

Furthermore, it goes without saying that all registers rely on accurate data collection and entry. In this context, quality protection in the ATR-DGU® is ensured through a high-quality certification process as well as regular, mandatory audits of all hospitals participating in the registry.

Finally, due to limitations of the standard documentation sheet thus far, only vitamin D supplementation and specific anti-osteoporotic therapy can be distinguished. A differentiation between bisphosphonates and other specific anti-osteoporotic medications cannot be made at the time. A possible revision of the standard documentation sheet could allow for a more precise statement on this issue in the future.

Despite these abovementioned limitations, due to the register design, data from a large number of patients suffering from ASFs, which are very rare overall, could be collected for this investigation. Additionally, the present study, which included patients from nearly 160 geriatric trauma centres throughout Germany, Switzerland and Austria, provides a comprehensive overview of the current treatment strategies and outcomes associated with ASFs in central Europe.

## Conclusions

The results of the present registry analysis represent a further building block in the current research on atypical femoral fractures. They revealed that patients with ASFs were more likely to be discharged home, died significantly less often and were more often treated with closed intramedullary reduction than patients with TSFs. An ASF pattern did not lead to increased rates of perioperative complications or prolonged reconvalescence, as indicated by insignificant rates in reoperation and walking abilities during follow-up.

### Electronic supplementary material

Below is the link to the electronic supplementary material.

Supplementary Material 1

## References

[1] WHO Study Group (1994). And its application to screening for postmenopausal osteoporosis. Report of a. World Health Organ Tech Rep Ser.

[2] Bliemel C, Sielski R, Doering B, Dodel R, Balzer-Geldsetzer M, Ruchholtz S (2016). Pre-fracture quality of life predicts 1-year survival in elderly patients with hip fracture-development of a new scoring system. Osteoporos Int.

[3] Aigner R, Meier Fedeler T, Eschbach D, Hack J, Bliemel C, Ruchholtz S (2016). Patient factors associated with increased acute care costs of hip fractures: a detailed analysis of 402 patients. Arch Osteoporos.

[4] Buecking B, Struewer J, Waldermann A, Horstmann K, Schubert N, Balzer-Geldsetzer M (2014). What determines health-related quality of life in hip fracture patients at the end of acute care?--a prospective observational study. Osteoporos Int.

[5] Bliemel C, Bieneck F, Riem S, Hartwig E, Liener UC, Ruchholtz S (2012). [Subsequent treatment following proximal femoral fracture - who, when, where? Assessment of the current situation in Germany]. Z Orthop Unfall.

[6] Bliemel C, Lechler P, Oberkircher L, Colcuc C, Balzer-Geldsetzer M, Dodel R (2015). Effect of Preexisting Cognitive Impairment on In-Patient treatment and Discharge Management among Elderly patients with hip fractures. Dement Geriatr Cogn Disord.

[7] Bliemel C, Buecking B, Oberkircher L, Knobe M, Ruchholtz S, Eschbach D (2017). The impact of pre-existing conditions on functional outcome and mortality in geriatric hip fracture patients. Int Orthop.

[8] Bliemel C, Oberkircher L, Eschbach DA, Lechler P, Balzer-Geldsetzer M, Ruchholtz S (2015). Impact of Parkinson’s disease on the acute care treatment and medium-term functional outcome in geriatric hip fracture patients. Arch Orthop Trauma Surg.

[9] Bliemel C, Buecking B, Hack J, Aigner R, Eschbach DA, Ruchholtz S (2017). Urinary tract infection in patients with hip fracture: an underestimated event?. Geriatr Gerontol Int.

[10] Hadji P, Klein S, Gothe H, Häussler B, Kless T, Schmidt T (2013). The epidemiology of osteoporosis–bone evaluation study (BEST): an analysis of routine health insurance data. Dtsch Arztebl Int.

[11] Lim SJ, Yeo I, Yoon PW, Yoo JJ, Rhyu KH, Han SB (2018). Incidence, risk factors, and fracture healing of atypical femoral fractures: a multicenter case-control study. Osteoporos Int.

[12] Schwabe U, Paffrath D, Ludwig W-D, Klauber J (12/2019). *Arzneiverordungs-Report 2019*: Springer Berlin Heidelberg

[13] Rizzoli R, Akesson K, Bouxsein M, Kanis JA, Napoli N, Papapoulos S (2011). Subtrochanteric fractures after long-term treatment with bisphosphonates: a European Society on clinical and economic aspects of osteoporosis and osteoarthritis, and International Osteoporosis Foundation Working Group Report. Osteoporos Int.

[14] Aspenberg P (2014). Denosumab and atypical femoral fractures. Acta Orthop.

[15] Takahashi M, Ozaki Y, Kizawa R, Masuda J, Sakamaki K, Kinowaki K (2019). Atypical femoral fracture in patients with bone metastasis receiving denosumab therapy: a retrospective study and systematic review. BMC Cancer.

[16] Giusti A, Hamdy NA, Papapoulos SE (2010). Atypical fractures of the femur and bisphosphonate therapy: a systematic review of case/case series studies. Bone.

[17] Shane E, Burr D, Abrahamsen B, Adler RA, Brown TD, Cheung AM (2014). Atypical subtrochanteric and diaphyseal femoral fractures: second report of a task force of the American Society for Bone and Mineral Research. J Bone Min Res.

[18] Adler RA, El-Hajj Fuleihan G, Bauer DC, Camacho PM, Clarke BL, Clines GA (2016). Managing osteoporosis in patients on long-term bisphosphonate treatment: report of a Task Force of the American Society for Bone and Mineral Research. J Bone Min Res.

[19] Dell RM, Adams AL, Greene DF, Funahashi TT, Silverman SL, Eisemon EO (2012). Incidence of atypical nontraumatic diaphyseal fractures of the femur. J Bone Min Res.

[20] Davenport D, Duncan J, Duncan R, Dick A, Bansal M, Edwards MR (2018). Outcomes for Elderly patients with atypical femoral fractures compared to typical femoral fractures for length of Stay, Discharge Destination, and 30-Day mortality rate. Geriatr Orthop Surg Rehabil.

[21] Gani L, Anthony N, Dacay L, Tan P, Chong LR, King TF (2021). Incidence of atypical femoral fracture and its mortality in a single Center in Singapore. JBMR Plus.

[22] Crouch G, Dhanekula ND, Byth K, Burn E, Lau SL, Nairn L (2021). The Sydney AFF score: a simple Tool to distinguish females presenting with atypical femur fractures Versus typical femur fractures. J Bone Min Res.

[23] Schilcher J, Michaëlsson K, Aspenberg P (2011). Bisphosphonate use and atypical fractures of the femoral shaft. N Engl J Med.

[24] Bliemel C, Rascher K, Knauf T, Hack J, Eschbach DA, Aigner R et al (2021) Early surgery does not improve outcomes for patients with periprosthetic femoral fractures-results from the Registry for Geriatric Trauma of the German Trauma Society. Med (Kaunas) 57(6). 10.3390/medicina57060517 10.3390/medicina57060517 PMC8224313 34064211

[25] Bliemel C, Rascher K, Oberkircher L, Schlosshauer T, Schoeneberg C, Knobe M et al (2022) Surgical Management and outcomes following pathologic hip fracture-results from a propensity matching analysis of the Registry for Geriatric Trauma of the German Trauma Society. Med (Kaunas) 58(7). 10.3390/medicina58070871 10.3390/medicina58070871 PMC9322375 35888590

[26] Bücking B, Hartwig E, Nienaber U, Krause U, Friess T, Liener U (2017). [Results of the pilot phase of the Age Trauma Registry DGU®]. Unfallchirurg.

[27] Shane E, Burr D, Ebeling PR, Abrahamsen B, Adler RA, Brown TD (2010). Atypical subtrochanteric and diaphyseal femoral fractures: report of a task force of the American Society for Bone and Mineral Research. J Bone Min Res.

[28] Kharazmi M, Hallberg P, Schilcher J, Aspenberg P, Michaëlsson K (2016). Mortality after atypical femoral fractures: a Cohort Study. J Bone Min Res.

[29] Khow KS, Paterson F, Shibu P, Yu SC, Chehade MJ, Visvanathan R (2017). Outcomes between older adults with atypical and typical femoral fractures are comparable. Injury.

[30] Prasarn ML, Ahn J, Helfet DL, Lane JM, Lorich DG (2012). Bisphosphonate-associated femur fractures have high complication rates with operative fixation. Clin Orthop Relat Res.

[31] Weil YA, Rivkin G, Safran O, Liebergall M, Foldes AJ (2011). The outcome of surgically treated femur fractures associated with long-term bisphosphonate use. J Trauma.

[32] Spanyer J, Barber LA, Lands H, Brown A, Bouxsein M, Heng M (2021). Health-related quality of life outcomes after surgical treatment of atypical femur fractures: a multicenter retrospective cohort study. JBMR Plus.

[33] Koh A, Guerado E, Giannoudis PV (2017). Atypical femoral fractures related to bisphosphonate treatment: issues and controversies related to their surgical management. Bone Joint J.

[34] Bogdan Y, Tornetta P, Einhorn TA, Guy P, Leveille L, Robinson J (2016). Healing Time and complications in Operatively treated atypical Femur fractures Associated with Bisphosphonate Use: a Multicenter Retrospective Cohort. J Orthop Trauma.

[35] Githens M, Garner MR, Firoozabadi R (2018). Surgical Management of atypical femur fractures Associated with Bisphosphonate Therapy. J Am Acad Orthop Surg.

[36] Bliemel C, Buecking B, Mueller T, Wack C, Koutras C, Beck T (2015). Distal femoral fractures in the elderly: biomechanical analysis of a polyaxial angle-stable locking plate versus a retrograde intramedullary nail in a human cadaveric bone model. Arch Orthop Trauma Surg.

[37] Spinelli MS, Marini E, Daolio PA, Piccioli A (2019) Atypical diaphyseal femoral fractures: Considerations on surgical technique. *Injury, 50 Suppl 2*, S65-S69, 10.1016/j.injury.2019.01.048 10.1016/j.injury.2019.01.048 30782394

[38] Rudran B, Super J, Jandoo R, Babu V, Nathan S, Ibrahim E (2021). Current concepts in the management of bisphosphonate associated atypical femoral fractures. World J Orthop.

[39] Everts-Graber J, Bonel H, Lehmann D, Gahl B, Häuselmann H, Studer U (2022). Incidence of atypical femoral fractures in patients on osteoporosis Therapy-A Registry-based Cohort Study. JBMR Plus.

[40] Kaku T, Oh Y, Sato S, Koyanagi H, Hirai T, Yuasa M (2020). Incidence of atypical femoral fractures in the treatment of bone metastasis: an alert report. J Bone Oncol.

[41] Omichi Y, Toki S, Nishisho T, Harada T, Sato N, Sairyo K (2023). Atypical femoral fracture in a multiple myeloma patient undergoing treatment with denosumab: a case report and literature review. Int J Surg Case Rep.

[42] Subramanian S, Parker M (2016) Atypical femur fractures - patient characteristics and results of intramedullary nailing for a series of 21 patients, vol 82. Acta Orthopaedica Belgica, pp 376–381 27682303

