# Supplementary figures and images for: Surgical management and outcomes following atypical subtrochanteric femoral fractures − results from a matched-pair analysis of the registry for geriatric trauma of the German Trauma Society
Source: Arch Orthop Trauma Surg. 2024 Apr 20;144(6):2561–72. doi: 10.1007/s00402-024-05297-3 (PMC11211164; doi:10.1007/s00402-024-05297-3)

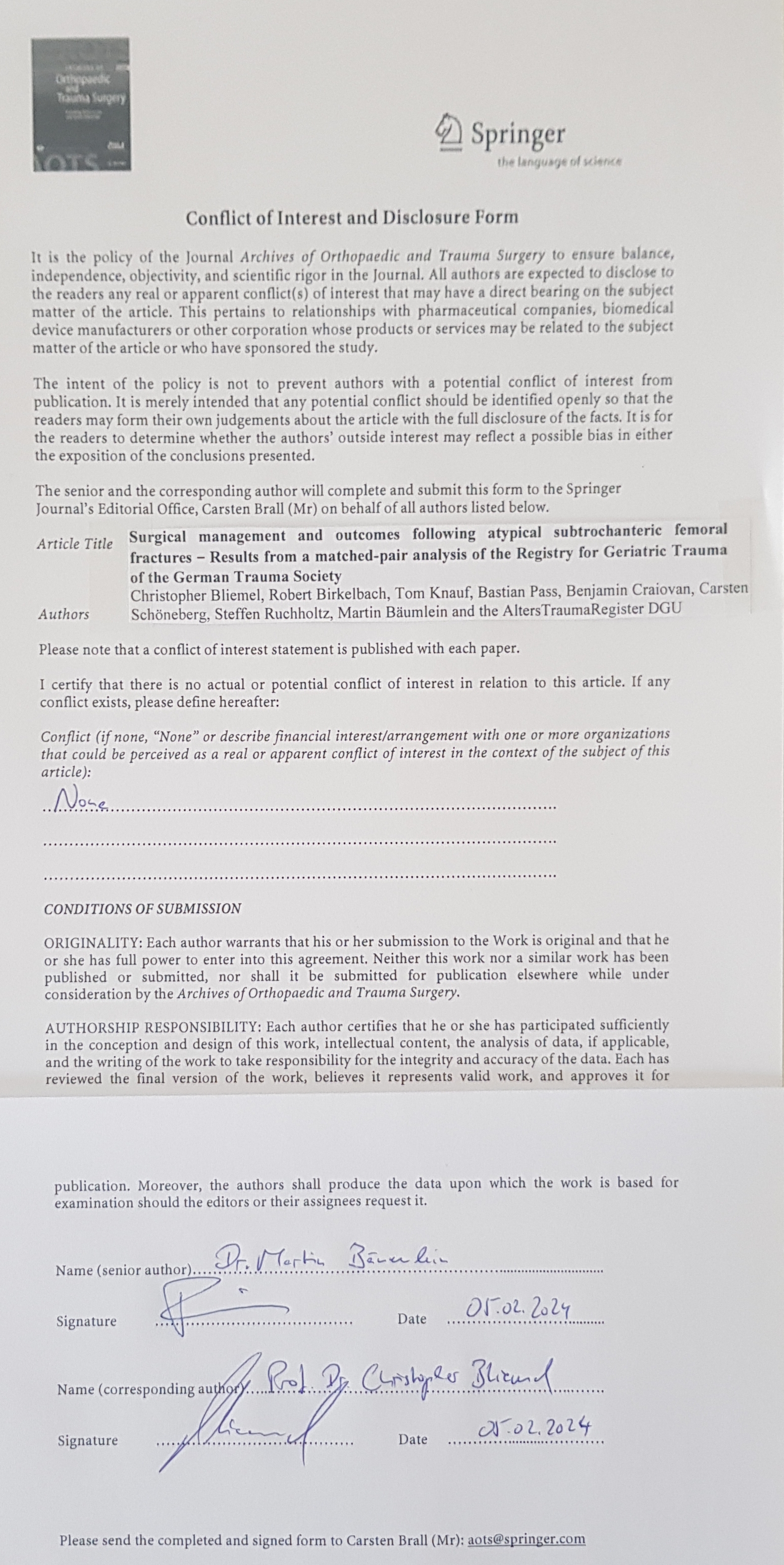

Supplement: Supplementary file 1 — Supplementary Material 1 [file 402_2024_5297_MOESM1_ESM.jpg]
